# The dorsal arcopallium of chicks displays the expression of orthologs of mammalian fear related serotonin receptor subfamily genes

**DOI:** 10.1038/s41598-020-78247-9

**Published:** 2020-12-03

**Authors:** Toshiyuki Fujita, Naoya Aoki, Chihiro Mori, Eiko Fujita, Toshiya Matsushima, Koichi J. Homma, Shinji Yamaguchi

**Affiliations:** 1 Faculty of Pharmaceutical Sciences, Department of Life and Health Sciences, Teikyo University, 2-11-1 Kaga, Itabashi-ku, Tokyo, 173-8605 Japan; 2 Department of Biology, Faculty of Science, Hokkaido University, Hokkaido, 060-0810 Japan

**Keywords:** Amygdala, Neural circuits, Neurochemistry

## Abstract

Fear is an adaptive emotion that elicits defensive behavioural responses against aversive threats in animals. In mammals, serotonin receptors (5-HTRs) have been shown to modulate fear-related neural circuits in the basolateral amygdala complex (BLA). To understand the phylogenetic continuity of the neural basis for fear, it is important to identify the neural circuit that processes fear in other animals. In birds, fear-related behaviours were suggested to be processed in the arcopallium/amygdala complex and modulated by the serotonin (5-HT) system. However, details about the distribution of 5-HTRs in the avian brain are very sparsely reported, and the 5-HTR that is potentially involved in fear-related behaviour has not been elucidated. In this study, we showed that orthologs of mammalian *5-HTR* genes that are expressed in the BLA, namely *5-HTR1A*, *5-HTR1B*, *5-HTR2A*, *5-HTR2C*, *5-HTR3A*, and *5-HTR4,* are expressed in a part of the chick arcopallium/amygdala complex called the dorsal arcopallium. This suggests that serotonergic regulation in the dorsal arcopallium may play an important role in regulating fear-related behaviour in birds. Our findings can be used as a basis for comparing the processing of fear and its serotonergic modulation in the mammalian amygdala complex and avian arcopallium/amygdala complex.

## Introduction

Serotonin (5-hydroxytryptamine, 5-HT) is a conserved modulatory neurotransmitter across vertebrate and invertebrate species^1^. In mammals, 5-HT is involved in a variety of physiological and behavioural functions, including processing of emotions^2^. In particular, the 5-HT system has long been implicated in the regulation of fear-related behaviours in mammals by modulating the neural circuits of the amygdala^3,4^. To improve the understanding of the phylogenetic continuity of the neural basis for emotions, it is important to elucidate the neural circuit that processes fear-related behaviours in animals other than mammals. Birds are animals that can potentially serve as models to analyse the control of fear-related reactions^5,6^. Indeed, lesions of the arcopallium/amygdala complex—a term that has been introduced by Herold et al*.*, to describe a region that combines arcopallium, the posterior pallial amygdala, the nucleus taeniae of the amygdala (TnA) and subpallial amygdaloid area^7^ —have been reported to reduce escape- and fear-related behaviour in birds^8–10^ and many reports have suggested that a large region of the arcopallium/amygdala complex could be partially homologous to the mammalian amygdala^11,12^. In addition, 5-HT/serotonin transporter systems have been suggested to modulate fear-related behaviours in chickens based on a combination of behavioural and functional polymorphisms^13–15^. However, because arcopallium/amygdala complex is a large and heterogenous brain region, the contribution of its individual subdivisions to the control of fear is unclear; furthermore, the neural circuits that are modulated by 5-HT for the fear processing in avian brains are largely unknown.

In mammals, the amygdala, which is comprised of a heterogeneous collection of nuclei derived from both the pallium and subpallium^11,12^, is a key brain region that is important for emotional processing, including the regulation of fear^16^. Under laboratory conditions, learned fear has been studied using Pavlovian fear conditioning^16,17^. In particular, the basolateral complex of the amygdala (BLA), which comprises the lateral (LA), basal (BA), and basomedial (BM) nuclei, has been well studied at the microcircuit level^17–19^. The LA receives sensory projections from outside the amygdala and projects to the BA, and the BA projects to the central nucleus of the amygdala (CeA) for output to the brainstem^17–19^. Every nucleus in the amygdala is densely innervated by fibres releasing the neurotransmitter 5-HT^20^. The released 5-HT acts on its downstream targets through multiple cell membrane receptors called 5-HT receptors (5-HTRs)^1^. To date, 14 distinct receptors have been classified into seven groups, namely 5-HTR1 to 5-HTR7, based on their structural, functional, and biochemical characteristics^1,21^. In the mammalian BLA, multiple 5-HTRs are expressed in various cell types, such as glutamatergic principal neurons and inhibitory interneurons^22^. Several 5-HTRs modulate the neurons that constitute the neural microcircuit that processes fear-related behaviours^22–28^.

On the contrary, to date, details about the distribution of 5-HTRs in avian brains are very sparsely reported, and the 5-HTR that is potentially involved in fear-related behaviour has not been elucidated. Therefore, the aim of the present study was to identify the potential 5-HTRs that are involved in the processing of fear-related behaviours in birds. In this study, we selected chick orthologs of six mammalian *5-HTR* genes and performed in situ hybridisation in the chick telencephalon. We found that all the used orthologs of *5-HTR*s were expressed in the dorsal arcopallium of chicks, which was proposed to be homologous to a part of mammalian BLA based on a detailed analysis of the embryonic origin and molecular profile (pattern of expression of morphogenetic genes during development)^11,12,29^. This result may support the proposed homology between dorsal arcopallium in birds and BLA in mammals. In addition, we found that *5-HTR2C* and *5-HTR4* were preferentially expressed in a part of the TnA, suggesting the serotonergic modulation of the TnA. Our findings can be used as a basis for understanding the serotonergic modulation in the mammalian amygdala complex and avian arcopallium/amygdala complex.

## Results

### Selection of the chick orthologs of mammalian *5-HTR* genes

We selected chick orthologs of six mammalian *5-HTR* genes. Five of these mammalian orthologs, that is, 5-*HTR1A, 5-HTR2A, 5-HTR2C, 5-HTR3A,* and *5-HTR4,* have been shown to be involved in the mammalian BLA microcircuit, which processes learned fear-related behaviour^22–28^. The mammalian ortholog of *5-HTR1B* is certainly expressed in the BLA, but it has not yet been shown to be associated with fear-related behaviour^30,31^. The orthologs exhibited the following sequence similarities (protein and DNA) between chicks and mice: *5-HTR1A*: 80.3% and 76.3%, respectively; *5-HTR1B*: 86.7% and 79.4%, respectively; *5-HTR2A*: 74.8% and 73.9%, respectively; *5-HTR3A*: 70.9% and 71.7%, respectively; *5-HTR4*: 84.3% and 80.5%, respectively. The following were the sequence similarities (protein and DNA) between chicks and tropical clawed frogs: *5-HTR2C*: 81.4% and 80.5%, respectively. These *5-HTR* characteristics are summarised in Table 1. When multiple transcript variants of the *5-HTR*s were registered in the database, probes were designed to detect all of them in this study. We performed in situ hybridisation and analysed the expression patterns in the orthologs in the whole telencephalon of the chick.

**Table 1:** Overview of the *5-HTR* genes used in this study.

Accession number	Gene symbol	Molecular characteristics	G Protein effector	Cellular response	Functional relations in the BLA studied using fear conditioning (mammalian orthologs)	References
NM_001170528.1	*5-HTR1A*	GPCR	Gi/o	inhibitory	promoting depression of EPSPs at synapses between LA PNs and BA PNs	Cheng, et al*.*^23^
XM_015284634.2	*5-HTR1B*	GPCR	Gi/o	inhibitory	–	–
XM_025151250.1	*5-HTR2A*	GPCR	Gq/11	excitatory	promoting induction of LTP at BA synapses via facilitated NMDA function, depolarization of GABAergic Ins in the BA	Chen, et al., Jiang, et al*.*^24,25^
XM_004940651.3	*5-HTR2C*	GPCR	Gq/11	excitatory	depolarising effect in LA PNs	Yamamoto, et al. 2014^26^
XM_004948063.3	*5-HTR3A*	ligand-gated ion channel	-	excitatory	depolarization of GABAergic INs in the BA	Rainnie, et al.^27^
XM_015293658.2	*5-HTR4*	GPCR	Gs	excitatory	LTP in the BA	Huang and Kandel^28^

### *5-HTR1A* expression in the chick pallium

**Figure 1:**
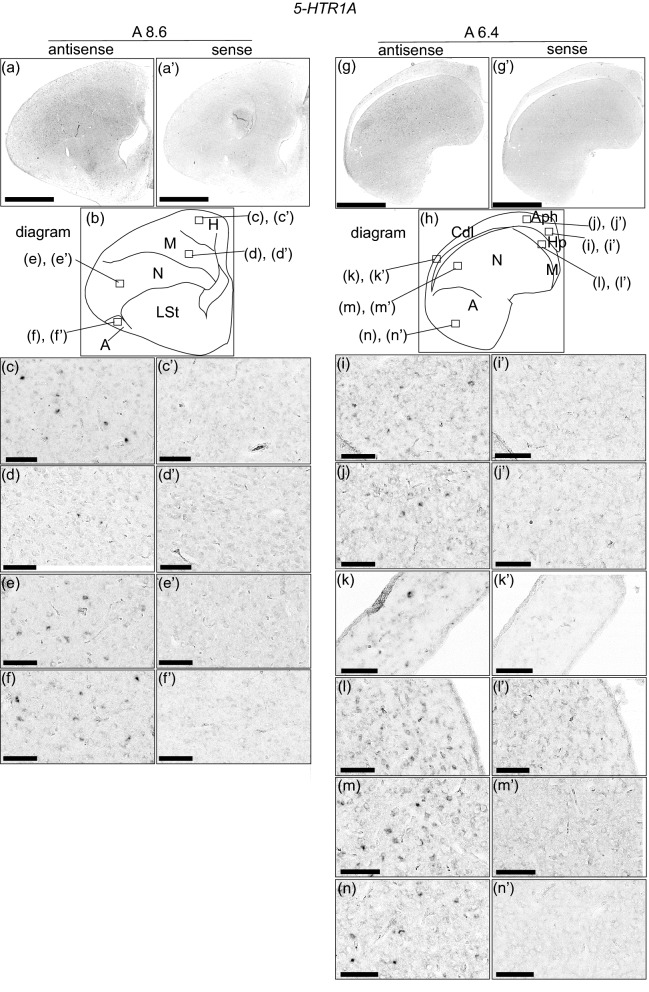
In situ hybridisation of 5-HTR1A in the P1 chick brains. DIG-labelled RNA antisense (**a**,**c**–**f**,**g**,**i**–**n**) and sense (**a’**,**c’**–**f’**,**g’**,**i’**–**n’**) *5-HTR1A* probe was used for in situ hybridisation in P1 chick brain coronal sections. To evaluate the expression patterns of *5-HTR1A*, sections of five chicks were analysed, and the representative levels of sections (A 8.6 and A 6.4) are shown. The levels of the sections are in accordance with the chick atlas by Kuenzel and Masson^32^. (**b**,**h**) Diagrams of coronal sections shown on panels (**a**) and (**g**), respectively. (**c**–**n**,**c’**–**n’**) Magnified views of brain areas are shown in the boxes in (**b**) and (**h**), respectively. *A* arcopallium; *Aph* area parahippocampalis; *Cdl* area corticoidea dorsolateralis; *H* hyperpallium; *Hp* hippocampus; *LSt* lateral striatum; *M* mesopallium; *N* nidopallium. Scale bars = 2.5 mm (**a**,**a’**,**g**,**g’**) and 100 µm (**c**–**f**), (**c’**–**f’**), (**i**–**n**), and (**i’**–**n’**).

We performed in situ hybridisation analysis to reveal the 5-*HTR1A* expression pattern in the naïve chick brains on post-hatched day 1 (P1). When we observed the entire sections of the brain hemisphere, we did not detect a significant signal from A 12.8 to A 5.8 (defined by Kuenzel and Masson atlas^32^) (Sup Fig. S1). However, when we observed each section of the brain in detail, we detected cells showing strong signals that were sparsely distributed throughout the pallium (Fig. 1). On coronal section A 8.6, signals were detected in the hyperpallium (Fig. 1c,c’), mesopallium (Fig. 1d,d’), nidopallium (Fig. 1e,e’), and arcopallium (Fig. 1f,f’). On coronal section A 6.4, signals were detected in the hippocampus (Hp) (Fig. 1i,i’), area parahippocampalis (APH) (Fig. 1j,j’), area corticoidea dorsolateralis (CDL) (Fig. 1k,k’), mesopallium (Fig. 1l,l’), nidopallium (Fig. 1m,m’), and arcopallium (Fig. 1n,n’).

### *5-HTR1B* expression in the chick pallium

**Figure 2:**
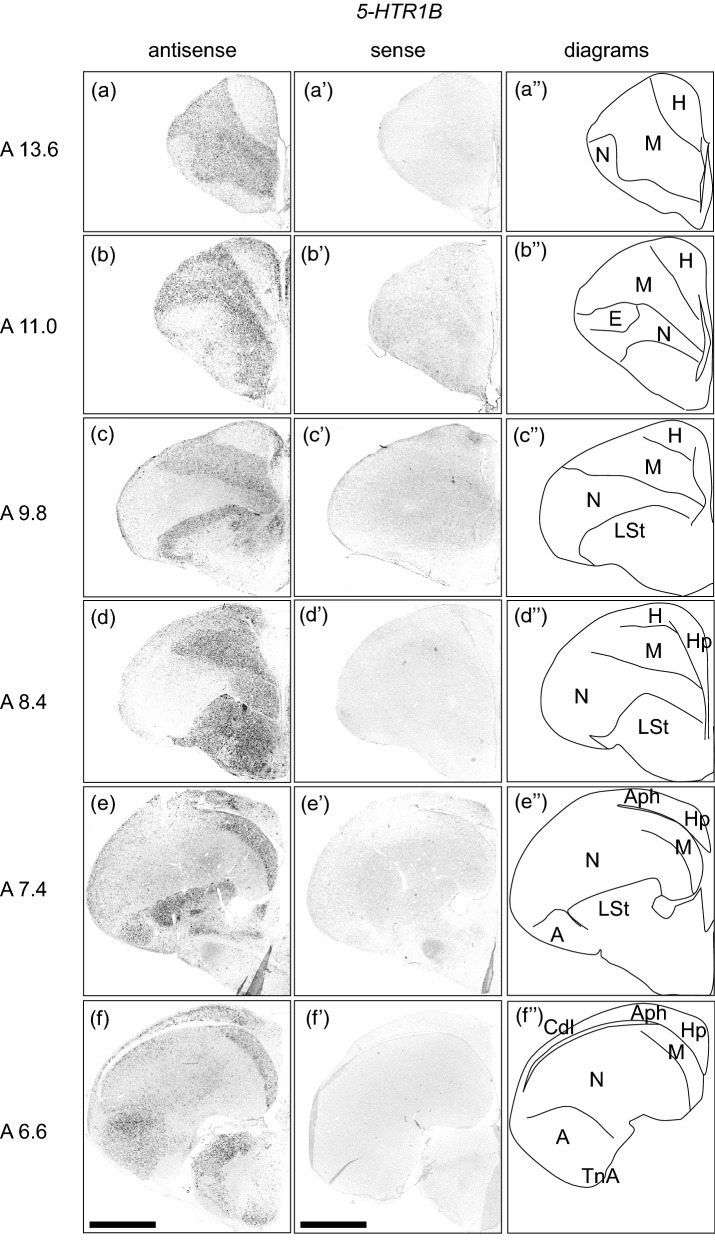
In situ hybridisation of 5-HTR1B in the P1 chick brains. DIG-labelled RNA antisense (**a**–**f**) and sense (**a’**–**f’**) *5-HTR1B* probes were used for in situ hybridisation in coronal sections of P1 chick brains. To evaluate the expression patterns of *5-HTR1B*, sections of eight chicks were analysed and representative images of four chick brain sections are shown. (**a’’**–**f’’**) Diagrams of coronal sections are shown on the rightmost panels. The levels of the sections (A 13.6 to A 6.6) are in accordance with the chick atlas by Kuenzel and Masson^32^. *A* arcopallium; *Aph* area parahippocampalis; *Cdl* area corticoidea dorsolateralis; *E* entopallium; *H* hyperpallium; *Hp* hippocampus; *LSt* lateral striatum; *M* mesopallium; *N* nidopallium; *TnA* nucleus taeniae of the amygdala. Scale bar = 2.5 mm.

We examined the expression level of 5-*HTR1B* in sections A 13.6 to A 6.6 in the P1 chick brains (Fig. 2). Strong signals were detected in the whole mesopallium (Fig. 2a–f’, A 13.6 to A 6.6), the medial nidopallium (Fig. 2b–d,b’–d’, A 11.0 to A 8.4), lateral nidopallium (Fig. 2e–f,e’–f’, A 7.4 to A 6.6), and the whole arcopallium (Fig. 2e–f,e’–f’, A 7.4 to A 6.6). In addition, sparse signals were detected in the hyperpallium (Fig. 2a–d,a’–d’, A 13.6 to A 8.4), hippocampus (Fig. 2d–f,d’–f’, A 8.4 to A 6.6), APH (Fig. 2e–f,e’–f’, A 7.4 to A 6.6), CDL (Fig. 2e–f,e’–f’, A 7.4 to A 6.6). Signals were detected in the striatum and subpallial amygdaloid area (Fig. 2c–f, c’–f’, A 9.8 to A 6.6).

### *5-HTR2A* expression in the chick pallium

**Figure 3:**
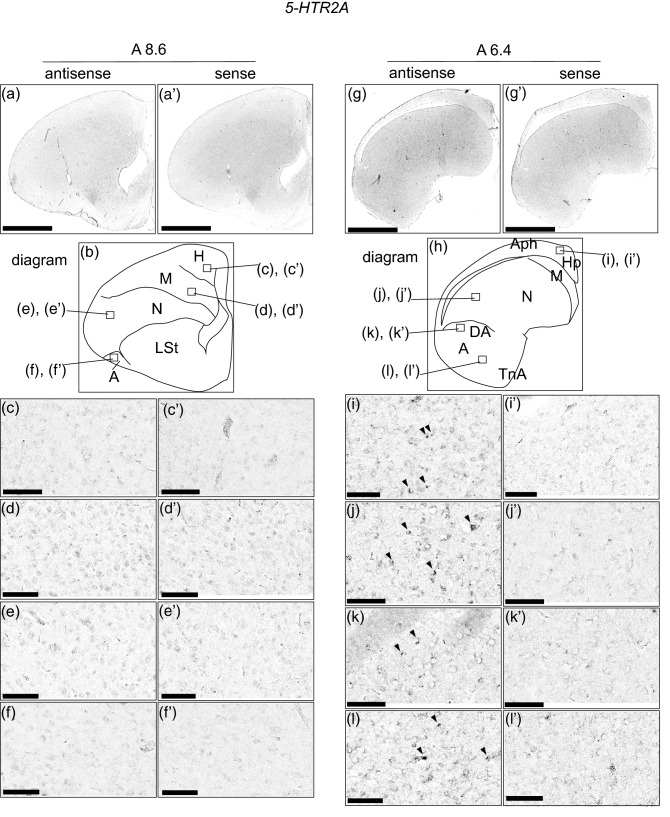
In situ hybridization of 5-HTR2A in the P1 chick brains. DIG-labelled RNA antisense (**a**,**c**–**f**,**g**,**i**–**l**) and sense (**a’**,**c’**–**f’**,**g’**,**i’**–**l’**) *5-HTR2A* probes were used for in situ hybridisation in coronal sections of P1 chick brains. To evaluate the expression patterns of *5-HTR2A*, sections of five chicks were analysed, and the representative levels of sections (A 8.6 and A 6.4) are shown. (**b**) and (**h**) Diagrams of coronal sections shown in panels (**a**) and (**g**), respectively. The levels of the sections (A 8.6 and A 6.4) are in accordance with the chick atlas by Kuenzel and Masson^32^. (**c**–**l**,**c’**–**l’**) Magnified views of brain areas shown in the boxes in (**b**) and (**h**), respectively. Arrowheads indicate signals. *A* arcopallium; *Aph* area parahippocampalis; *DA* dorsal arcopallium; *H* hyperpallium; *Hp* hippocampus; *M* mesopallium; *N* nidopallium; *TnA* nucleus taeniae of the amygdala. Scale bars = 2.5 mm (**a**,**a’**,**g**,**g’**) and 100 µm (**c**–**f**), (**c’**–**f’**), (**i**–**l**), (**i’**–**l’**).

We examined the expression level of 5-*HTR2A* in sections A 13.6 to A 5.8 and did not detect clear expression patterns when we observed the entire brain hemisphere sections (Sup Fig. S2). However, when we observed each brain region of the sections in detail (Fig. 3), we detected cells showing signals in the hippocampus (Fig. 3i,i’), lateral nidopallium (Fig. 3j,j’), dorsal arcopallium (Fig. 3k,k’), and intermediate arcopallium (Fig. 3l,l’) in section A 6.4 alone. We did not detect any signals in the remaining sections.

### *5-HTR2C* expression in the chick pallium

**Figure 4:**
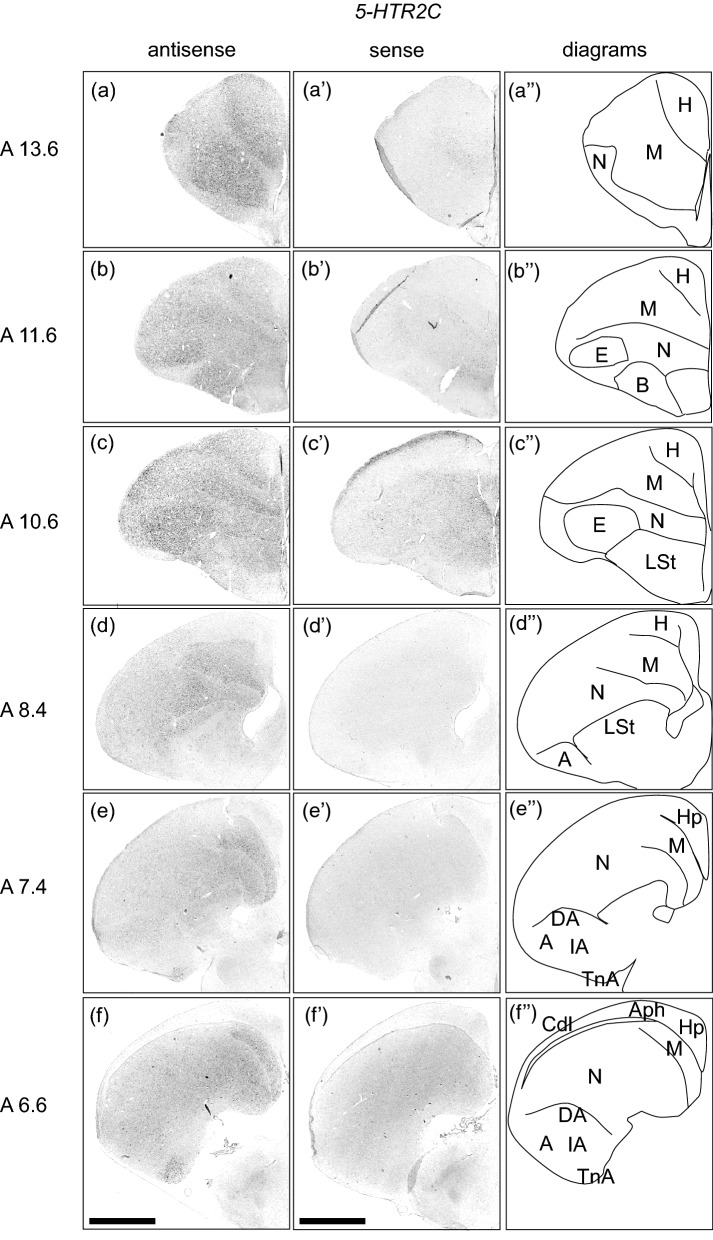
In situ hybridisation of 5-HTR2C in the P1 chick brains. DIG-labelled RNA antisense (**a**–**f**) and sense (**a’**–**f’**) *5-HTR2C* probes were used for in situ hybridisation in coronal sections of P1 chick brains. To evaluate the expression patterns of *5-HTR2C*, sections of seven chicks were analysed and representative images of three chick brain sections are shown. (**a’’**–**f’’**) Diagrams of coronal sections are shown on the rightmost panels. The levels of the sections (A 13.6 to A 6.6) are in accordance with the chick atlas by Kuenzel and Masson^32^. *A* arcopallium; *Aph* area parahippocampalis; *B* basorostralis; *Cdl* area corticoidea dorsolateralis; *DA* dorsal arcopallium; *E* entopallium; *H* hyperpallium; *Hp* Hippocampus; *IA* intermediate arcopallium; *LSt* lateral striatum; *M* mesopallium; *N* nidopallium; *TnA* nucleus taeniae of the amygdala. Scale bar = 2.5 mm.

We examined the expression level of 5-*HTR2C* in sections A 13.6 to A 6.6 in the P1 chick brains (Fig. 4). Strong signals were detected in the ventral and dorsal mesopallium (Fig. 4a–c,a’–c’, A 13.6 to A 10.6), entopallium (Fig. 4b–c,b’–c’, A 11.6 to A 10.6), basorostralis (Fig. 4b,b’, A 11.6), field L (Fig. 4e–f,e’–f’, A 7.4 to A 6.6), and TnA (Fig. 4e–f,e’–f’, A 7.4 to A 6.6). In addition, relatively weak signals were detected in almost the entire nidopallium (Fig. 4a–f,a’–f’, A 13.6 to A 6.6), the dorsal and intermediate arcopallium (Fig. 4e–f,e’–f’, A 7.4 to A 6.6), and the striatum and subpallial amygdaloid area (Fig. 2d–e,d’–e’, A 8.4 to A 7.4).

### *5-HTR3A* expression in the chick pallium

**Figure 5:**
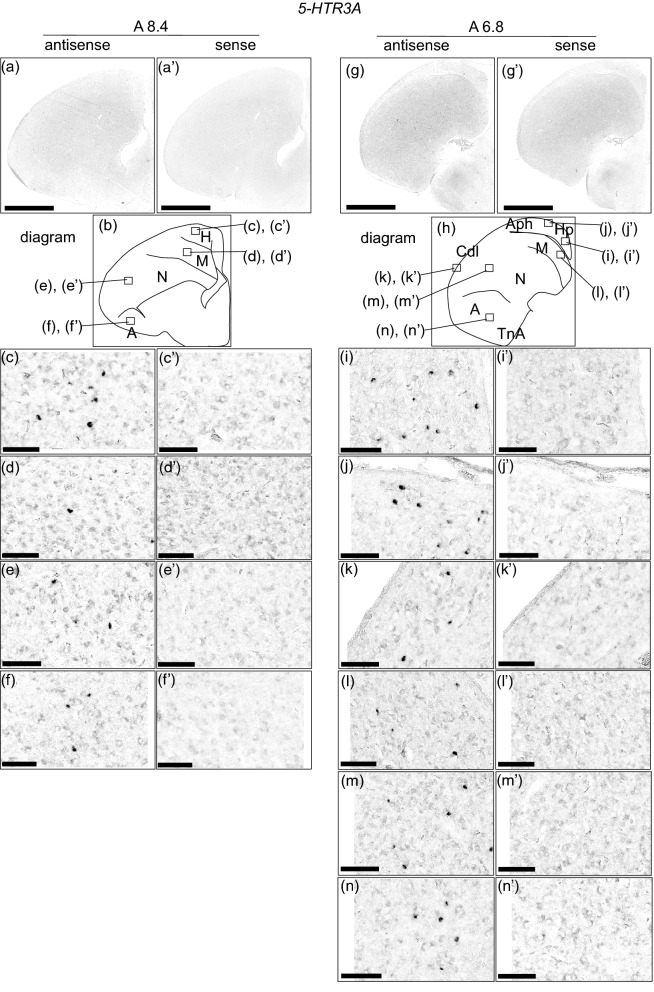
In situ hybridisation of 5-HTR3A in the P1 chick brains. DIG-labelled RNA antisense (**a**,**c**–**f**,**g**,**i**–**n**) and sense (**a’**,**c’**–**f’**,**g’**,**i’**–**n’**) *5-HTR3A* probe was used for in situ hybridisation in P1 chick brain coronal sections. To evaluate the expression patterns of *5-HTR3A*, sections of eight chicks were analysed, and representative images of chick brain sections are shown. (**b**) and (**h**) Diagrams of coronal sections shown on panels (**a**) and (**g**), respectively. Representative levels of sections (A 8.6 and A 6.4) are shown. The levels of the sections are in accordance with the chick atlas by Kuenzel and Masson^32^. (**c**–**n**,**c’**–**n’**) Magnified views of brain areas shown in the boxes in (**b**) and (**h**), respectively. *A* arcopallium; *Aph* area parahippocampalis; *Cdl* area corticoidea dorsolateralis; *H* hyperpallium; *Hp* hippocampus; *M* mesopallium; *N* nidopallium; *TnA* nucleus taeniae of the amygdala. Scale bars = 2.5 mm (**a**,**a’**,**g**,**g’**) and 100 µm (**c**–**f**), (**c’**–**f’**), (**i**–**n**), (**i’**–**n’**).

A clear expression pattern was not detected when we observed the entire sections of brain hemisphere (Sup Fig. S3a–f’). When we observed each brain region of the sections in detail, cells showing strong signals were detected in the whole pallium (Fig. 5). In section A 8.4, cells showing strong signals were detected in the hyperpallium (Fig. 5c,c’), mesopallium (Fig. 5d,d’,l,l’), nidopallium (Fig. 5e,e’,m,m’), and arcopallium (Fig. 5f,f’,n,n’). In section A 6.8, cells showing strong signals were detected in the hippocampus (Fig. 5i,i’), APH (Fig. 5j,j’), and CDL (Fig. 5k,k’).

### *5-HTR4* expression in the chick pallium

**Figure 6:**
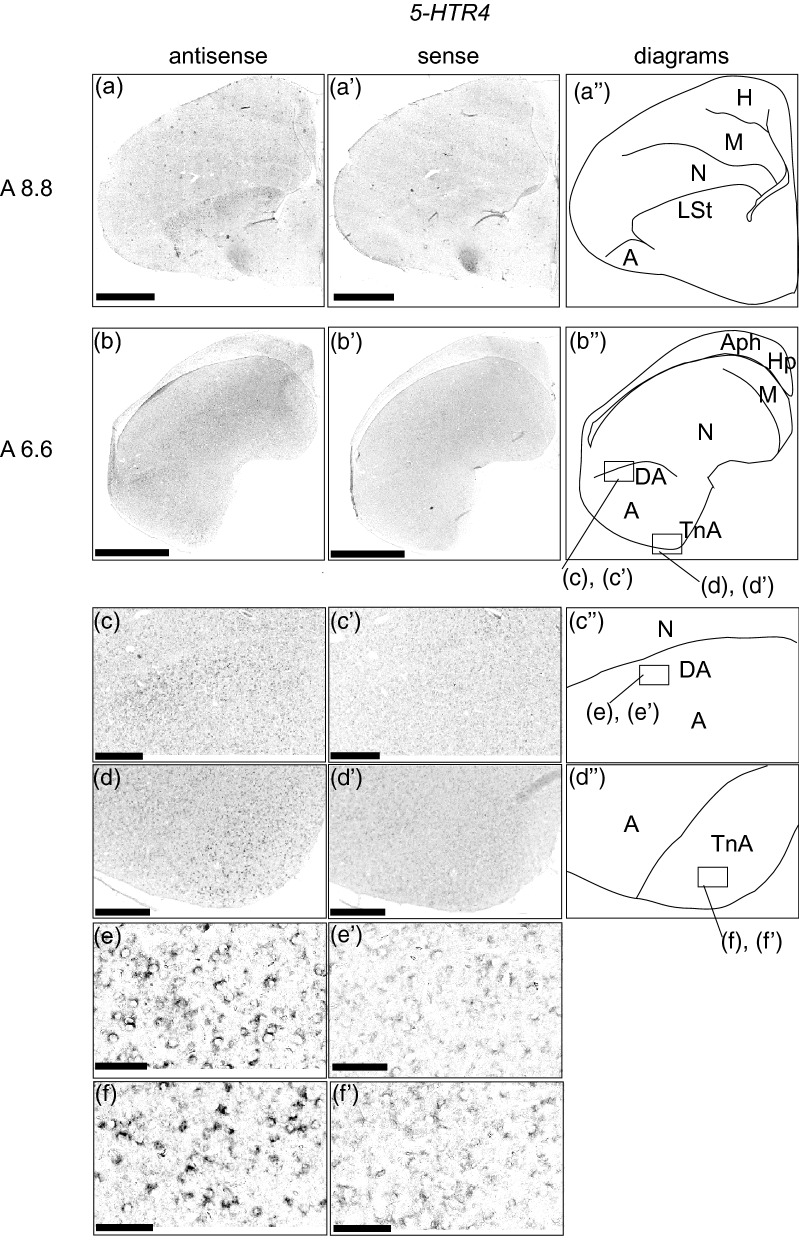
In situ hybridization of 5-HTR4 in the P1 chick brains. DIG-labelled RNA antisense (**a**–**f**) and sense (**a’**–**f’**) *5-HTR4* probes were used for in situ hybridisation in P1 chick brain coronal sections. To evaluate the expression patterns of *5-HTR4*, sections of six chicks were analysed, and a representative image is shown. (**a’’**–**d’’**) Diagrams of coronal sections are shown on the rightmost panels. The level of the sections (A 8.8 and A 6.6) is in accordance with the chick atlas by Kuenzel and Masson^32^. (**c**,**d**,**c’**,**d’**) Magnified views of brain areas are shown in the boxes in (**b’’**). (**e**,**f**,**e’**,**f’**) Magnified views of brain areas shown in the boxes in (**c’’**) and (**d’’**). *A* arcopallium; *Aph* area parahippocampalis; *DA* dorsal arcopallium; *Hp* hippocampus; *LSt* lateral striatum; *M* mesopallium; *N* nidopallium; *TnA* nucleus taeniae of the amygdala. Scale bars = 2.5 mm (**a**,**b**,**a’**,**b’**), 500 µm (**c**,**d**,**c’**,**d’**), and 100 µm (**e**,**f**,**e’**,**f’**).

We examined the expression of 5-*HTR4* in sections A 13.6 to A 5.6 (Sup Fig. S4) and detected signals in sections A 8.8 and A 6.6 (Fig. 6). We detected signals in the lateral striatum (LSt) (Fig. 6a,a’) in section A 8.8 and in the dorsal arcopallium (Fig. 6b,c,e,b’,c’,e’) and TnA (Fig. 6b,d,f,b’,d’,f’) in section A 6.6. We could not detect significant signals in the remaining sections.

### Comparison of the *5-HTRs*-expression patterns in the arcopallium/amygdala complex region

**Figure 7:**
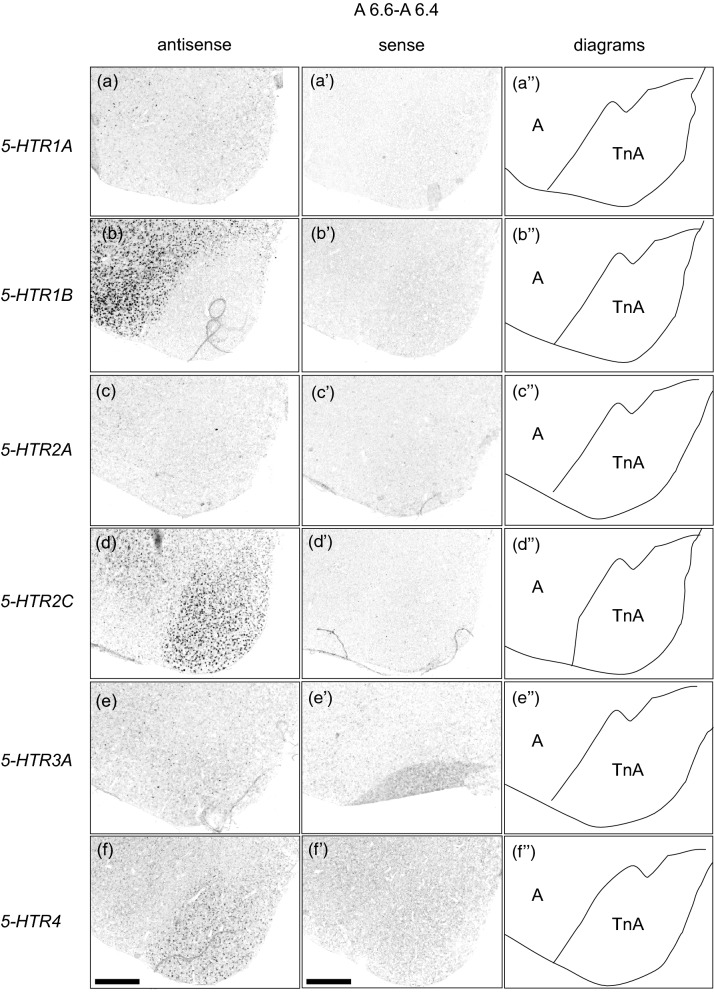
Comparison of the *5-HTRs*-expression patterns in the arcopallium/amygdala complex in the P1 chick brains using neighboring sections. In situ hybridisation using DIG-labelled RNA antisense and sense *5-HTR1A* (**a**) and (**a’**), *5-HTR1B* (**b**) and (**b’**), 5-*HTR2A* (**c**) and (**c’**), *5-HTR2C* (**d**) and (**d’**), *5-HTR3A* (**e**) and (**e’**), and *5-HTR4* (**f**) and (**f’**) probes in coronal sections of P1 chick brains. (**a’’**–**f’’**) Diagrams of coronal sections are shown in the rightmost panels, respectively. A, arcopallium; TnA, nucleus taeniae of the amygdala. Scale bars = 500 µm.

**Figure 8:**
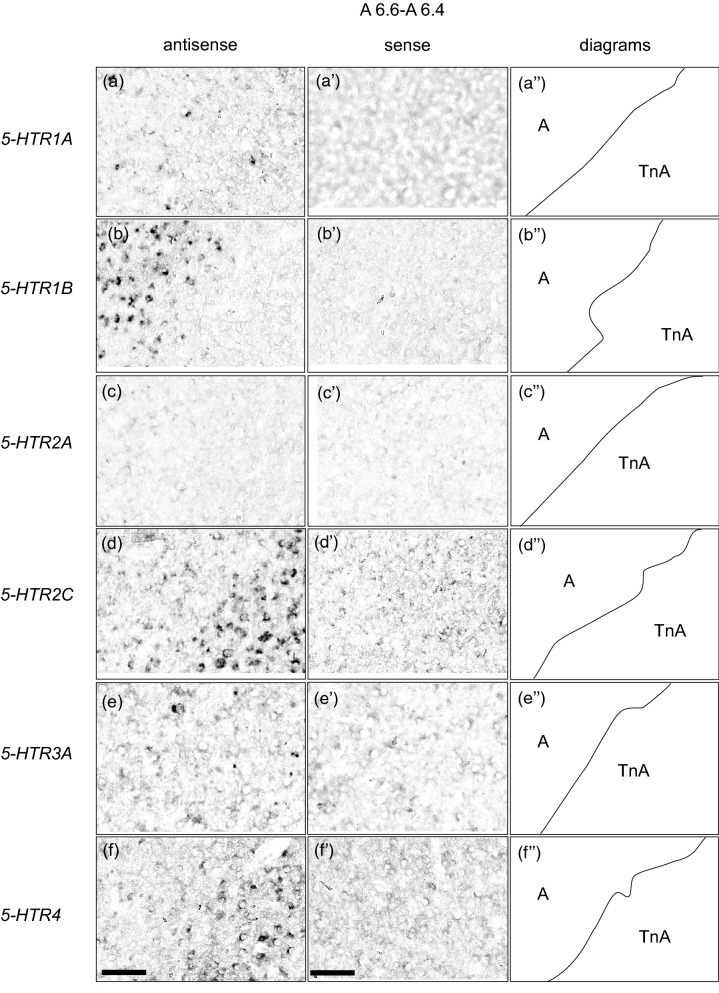
Comparison of the *5-HTRs*-expression patterns in the arcopallium/amygdala complex in the P1 chick brains using neighboring sections. Magnified views of the regions around the arcopallium and TnA boundaries are shown in Fig. 7a–f’. In situ hybridisation using DIG-labelled RNA antisense and sense *5-HTR1A* (**a**) and (**a’**), magnified view of Fig. 7a,a’, *5-HTR1B* (**b**) and (**b’**), magnified view of Fig. 7b,b’, 5-*HTR2A* (**c**) and (**c’**), magnified view of Fig. 7c,c’, and *5-HTR2C* (**d**) and (**d’**), magnified view of Fig. 7d,d’, *5-HTR3A* (**e**) and (**e’**), magnified view of Fig. 7e,e’, and *5-HTR4* (**f**) and (**f’**), magnified view of Fig. 7f,f’ probes in coronal sections of P1 chick brains. (**a’’**–**f’’**) Diagrams of coronal section are shown in the rightmost panels. A, arcopallium; TnA, nucleus taeniae of the amygdala. Scale bars = 100 µm.

All the examined 5-*HTRs* were expressed in the arcopallium/amygdala complex region as mentioned above. Subsequently, we compared the expression patterns of these HTRs in the arcopallium/amygdala complex region by using neighboring sections around A 6.6 to A 6.4 (Figs. 7 and 8). Cells showing *5-HTR1A* signals were detected sparsely in both the arcopallium and TnA (Fig. 7a,a’, Fig. 8a,a’). *5-HTR1B* signals were detected in the whole arcopallium, but no signal was detected in the TnA (Fig. 7b,b’, 8b,b’). *5-HTR2A* signals were detected weakly in a part of the arcopallium but not in the TnA (Figs. 7c,c’, 8c,c’). *5-HTR2C* signals were detected in the intermediate arcopallium and whole TnA (Figs. 7d,d’, 8d,d’). Cells with *5-HTR3A* signals were detected sparsely and thoroughly in both the arcopallium and TnA (Figs. 7e,e’, 8e,e’). *5-HTR4* signals were detected in the whole TnA and not detected the TnA adjacent area of the arcopallium (Figs. 7f, f’, 8f,f’).

## Discussion

**Figure 9:**
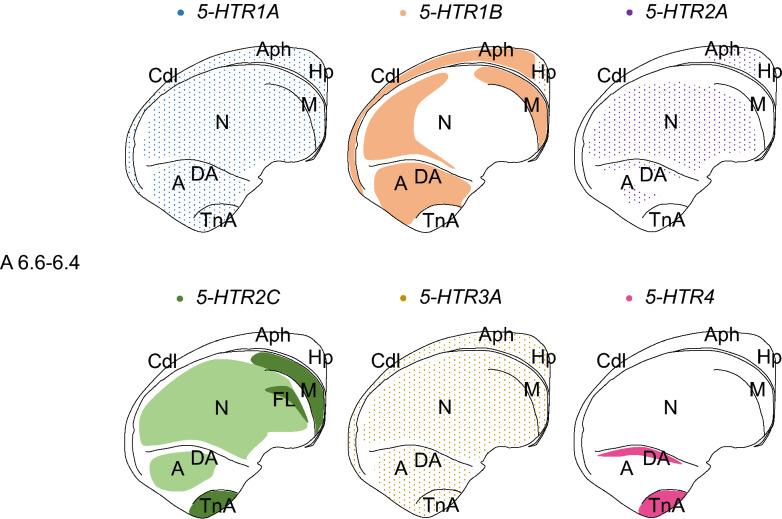
Schematic summary of the expression patterns of the six 5-HTRs (*5-HTR1A*, *5-HTR1B*, *5-HTR2A*, *5-HTR2C*, *5-HTR3A*, *5-HTR4*) in P1 chicks. Representative expression patterns in sections around A 6.6 to A 6.4 are represented by coloured areas (blue, *5-HTR1A*; orange, *5-HTR1B*; purple, *5-HTR2A*; green, *5-HTR2C*; yellow, *5-HTR3A*; magenta, *5-HTR4*). The darker the colour, the higher the level of gene expression. The dot pattern indicates that the expressed cells are distributed sparsely. The levels of the sections are in accordance with the chick atlas by Kuenzel and Masson^32^. *A* arcopallium; *Aph* area parahippocampalis; *Cdl* area corticoidea dorsolateralis; *DA* dorsal arcopallium; *FL* field L; *Hp* hippocampus; *M* mesopallium; *N* nidopallium; *TnA* nucleus taeniae of the amygdala.

In the present study, we revealed the expression patterns of six orthologs of mammalian *5-HTRs*, that is, *5-HTR1A*, *5-HTR1B*, *5-HTR2A*, *5-HTR2C*, *5-HTR3A*, and *5-HTR4*, in the telencephalon of chicks (Fig. 9). Previous studies have shown that all mammalian 5-HTRs orthologs are expressed in the mammalian BLA and are associated with fear processing in the rodent BLA, except for *5-HTR1B*^22–28,30,31^. In this study, we showed that all the used orthologs of *5-HTR*s were expressed in the dorsal arcopallium of chicks, which was proposed to be homologous to a part of mammalian BLA based on a detailed analysis of the embryonic origin and molecular profile (pattern of expression of morphogenetic genes during development) (Fig. 9)^11,12,29^; this suggests that the role of the dorsal arcopallium in fear-related behaviour is similar to that of the mammalian BLA. The arcopallium is a large, heterogenous brain region, but we are beginning to understand the contribution of its individual subdivisions to the regulation of fear-related behaviours^10^. Our findings support the proposal that the dorsal arcopallium is homologous to a part of the BLA based on developmental studies^11,12,29^.

The lateral nidopallium is also proposed to be partially homologous to the BLA based on a detailed analysis of the embryonic origin and gene expression profile^11,12,29^. Our data showed that *5-HTR4* was preferentially expressed in the dorsal arcopallium (Figs. 6b,b’ and Fig. 9) and that *5-HTR1B* was expressed more abundantly in the dorsal arcopallium than in the lateral nidopallium (Figs. 2f,f’ and Fig. 9). This evidence suggests that the avian dorsal arcopallium and lateral nidopallium have an innervation by serotonergic fibres in common with the mammalian BLA, but the avian dorsal arcopallium resembles the mammalian BLA more closely than does the lateral nidopallium in terms of serotonergic modulation. In the absence of a neocortex in birds, the lateral nidopallium is often considered functionally analogous (but not homologous) to the prefrontal cortex of mammals because of its extensive reciprocal connections with many pallial areas; this implies an important role of the lateral nidopallium as a highly associative centre related to cognition^33,34^. The lateral arcopallium is another region that is suggested to be functionally and anatomically analogous to the mammalian BLA^35^. Similarly, *5-HTR1B* was expressed less abundantly in the lateral arcopallium, and other *5-HTRs* were expressed sparsely in the lateral arcopallium (Figs. 2f,f’ and Fig. 9), suggesting that the lateral arcopallium was modulated by 5-HT and that the avian dorsal arcopallium resembles the mammalian BLA more closely than does the lateral arcopallium in terms of serotonergic modulation. Collectively, our findings imply that the avian dorsal arcopallium is not only homologous but also functionally analogous to the mammalian BLA. Indeed, this notion is consistent with recent findings that the mammalian BLA correlates with the reptilian anterior dorsal ventricular ridge, a part of which is the counterpart of avian arcopallium, using single-cell transcriptome^36^.

We also found that 5*-HTR1A*, *5-HTR2C*, *5-HTR3A*, and *5-HTR4* were expressed in the TnA, whereas *5-HTR1B* and *5-HTR2A* were not expressed in the TnA but in the region of the arcopallium just adjacent to the TnA. The TnA mostly corresponds to the subpallial medial amygdala of mammals^37–40^. Similar to its mammalian counterpart, this region is functionally associated with a variety of social behaviours such as sexual behaviours^41^ and social interactions at large^42,43^. Our findings regarding the expression patterns of *5-HTR*s in the TnA indicated the possibility that 5-HT plays a key role in shaping social responses. Because *5-HTR2C* and *5-HTR4* were preferentially expressed in the TnA, analysing the roles of 5-HTR2C and 5-HTR4 in the TnA may lead to an understanding of the importance of 5-HT in social responses in birds. In this study, we used the Kuenzel and Masson atlas^32,37^ to determine the location of TnA although the precise location of the TnA in the avian brains is not consistent^38,40,43^. The location of the TnA was defined differently in the chicken atlases by Puelles et al.^44^ and the Kuenzel and Masson atlas^32^.

As for the expression pattern of *5-HTR1A*, cells expressing it were sparsely distributed throughout the chick pallium, including the hyperpallium, mesopallium, nidopalliuim, arcopallium, Hp, APH, CDL. There was no difference in the density of *5-HTR1A-*expressing neurones between the intra-pallial regions. However, previous studies using radioligand autoradiography revealed that the 5-HTR1A densities in the nidopallium and hyperpallium were higher than those in the Hp and arcopallium/amygdala complex in the pigeon pallium^7,45^. The difference in the 5-HTR1A signal density between the intra-pallial regions may reflect the density of the axonal terminals of 5-HT neurones projected from the midbrain. Normally, mRNA in the axonal terminals cannot be detected by in situ hybridisation, but receptor binding sites were detected both in the pre- and post-synaptic neurones using radioligand autoradiography^30^.

As for the expression pattern of *5-HTR1B*, it was expressed in the whole mesopallium, arcopallium, striatum, and subpallial amygdaloid area and a part of hyperpallium, nidopallium, Hp, APH, CDL. In mammals, 5-HTR1B is distributed mainly in the basal ganglia regions and several cortical areas; it is also distributed in the amygdala^30^. Consistent with the mammalian 5-HTR1B distribution, the *5-HTR1B* expression levels in the subpallial region seemed to be most densely distributed in the chick brain, suggesting the conserved modulatory function of 5-HTR1B in the striatum and subpallial amygdaloid area.

As for the expression pattern of *HTR2A* in rats, it was abundantly expressed in the prefrontal cortex, all other cortical areas, and other areas including the amygdala^30,46,47^. We could not detect the *5-HTR2A* signals in the chicken hyperpallium, which is considered to be homologous to the mammalian neocortex^48,49^. This could have occurred because of the diversity of architecture between mammals and birds.

As for the expression pattern of *5-HTR2C*, it is majorly expressed in the intercalated nidopallium (consisting of the basorostralis, entopallium, and field L^50^) and TnA in chickens. The intercalated nidopallium receives sensory projections from the thalamus, which suggests that serotonergic modulation through 5-HTR2C plays an important role in sensory input processing in the intercalated nidopallium in birds. In terms of TnA expression, high expression levels of *5-HTR2C* have been observed in the rodent medial amygdala, which is considered to be the counterpart of the avian TnA^51,52^, suggesting the conserved roles of 5-HTR2C in the mammalian medial amygdala and avian TnA. For example, exposure to novel objects or novel environments is associated with the activation of TnA in avian species^53,54^. Avian species exhibit a reluctance to approach novel objects or novel environments (neophobia). Analysing serotonergic modulation via 5-HTR2C in TnA may lead to an understanding of the responsive neural circuits for the processing of neophobia, the fear-related responses to novelty in birds.

As for the expression pattern of *5-HTR3A*, cells expressing it were distributed throughout the chick pallium, including the hyperpallium, mesopallium, nidopallium, arcopallium, Hp, APH, and CDL. Similarly, in mammals, 5-HTR3A expressing cells were distributed sparsely throughout the brain including the cortical areas^30,55^. In addition, many of the *5-HTR3A-*expressing cells were shown as GABAergic interneurons. Especially in the amygdala, all *5-HTR3A-*expressing cells were GABAergic^30,56^. Considering the similarity in the distribution patterns of mammalian and avian *5-HTR3A*-expressing cells, we assumed that *5-HTR3A*-expressing cells in avian arcopallium/amygdala complex are most likely to be GABAergic interneurons.

As for the expression pattern of *5-HTR4*, it was expressed in the LSt, dorsal arcopallium, and TnA. In several mammalian species, it was revealed that the 5-HTR4-enriched cells are mainly distributed in the basal ganglia, except the globus pallidus and substantia nigra, and distributed in many pallial areas, including the amygdala^30,57^. Regarding the basal ganglia, similarities in the expression patterns of *5-HTR4* between mammals and birds suggest that the basal ganglia are subject to conserved serotonergic modulation via 5-HTR4.

In this study, we mainly focused on the control of fear-related behaviour as a physiological function in which the 5-HT system is potentially involved with in birds. Besides the regulation of fear-related behaviours by modulating the neural circuits of the amygdala, the 5-HT system is involved in a variety of physiological functions such as sleep, appetite, sensory processing, locomotor activity, cognition, and emotion in mammals^2^. We have shown that the *5-HTR*s are found in a variety of brain regions other than the avian arcopallium/amygdala complex (Fig. 9). These other brain regions may be involved in the regulation of a variety of physiological and behavioural functions by modulating the neural circuits in birds.

In summary, we found that all the *5-HTR*s used in this study were expressed in the dorsal arcopallium, which was proposed to be homologous to a part of the mammalian BLA^11,12,29^. This result may support the proposed homology between the dorsal arcopallium in birds and the BLA in mammals. In addition, we found that *5-HTR2C* and *5-HTR4* were preferentially expressed in a part of the TnA, suggesting the serotonergic modulation of the TnA. Our findings can be used as a basis for understanding the serotonergic modulation in the mammalian amygdala complex and avian arcopallium/amygdala complex.

## Methods

### Animals

Fertilised eggs of domestic chicks (*Gallus gallus domesticus*, the Cobb strain) were purchased from a local dealer (3-M, Aichi, Japan). Eggs were incubated at Teikyo University (Kaga, Itabashi-ku, Tokyo). Animal experiments were carried out as described previously^58,59^. Newly hatched chicks (P0) were transferred to dark plastic enclosures in a dark warm cage at 30 °C for one day (P1). In this study, we used 5 chicks for the *HTR1A* condition, 8 for the *HTR1B* condition, 5 for the *HTR2A* condition, 7 for the *HTR2C* condition, 8 for the *HTR3A* condition, and 6 for the *HTR4* condition (a total of 11 chicks). We summarized the information about the number of animals we used in this study in Supplemental Table S1. All procedures were reviewed and approved by the Committee on Animal Experiments of Teikyo University and conducted according to the guidelines of the national regulations for animal welfare in Japan.

### Histological procedures

First, P1 chicks were deeply anaesthetised by intraperitoneal injection (0.40 mL/individual) of 1:1 solution of ketamine (10 mg/mL, ketalar-10, Sankyo Co., Tokyo, Japan) and xylazine (2 mg/mL, Sigma, St. Louis, Missouri, USA). Then, the chicks were transcardially perfused with 4% paraformaldehyde in 0.1 M phosphate buffered saline (pH 7.5) (PFA-PBS). Dissected brains were immersed in PFA-PBS overnight at 4 °C and placed in an 18% sucrose/PFA-PBS solution for cryoprotection for two days at 4 °C. Subsequently, brains with sucrose substitution were embedded in Tissue-Tek OCT compound (Sakura Finetechnical, Tokyo, Japan), frozen immediately on dry ice, and stored at − 80 °C until use.

### cDNA cloning and RNA probe preparation

Total RNA was extracted from the chick brain using TRIzol Reagent (Invitrogen, Carlsbad, CA, USA) and reverse-transcribed with SuperScript III kit (Invitrogen, Carlsbad, CA, USA) using an oligo (dT) primer, according to the manufacturer’s protocol. RT-PCR was performed using the following gene specific primer (forward and reverse) pairs: *HTR1A:* 5′-AACACTACCTCCCCAGAACG-3′ and 5′-TCCCCGTTGCTTTTCTTCTG-3′, respectively; *HTR1B:* 5′-TCACGTGGCTGGGATATCTC-3′ and 5′-CACTGCCACTTCTCACACAC-3′, respectively; *HTR2A:* 5′-GTGTTTAAGAAAGGCCACTGC-3′ and 5′-TTCCCTGGACTGATGCTTCC-3′, respectively; *HTR2C:* 5′-CTGACTGTCCAGGTGCTACA-3′ and 5′-TCCATTGACCAACGCTTACA-3′, respectively; *HTR3A:* 5′-TCCAGAACCTCAAGCCCATC-3′ and 5′-CCGTAGTGGTTCAGTTTGGC-3′, respectively; *HTR4:* 5′-ATGTGAGTTCGAGTGAGGGC-3′ and 5′-GTCTTGGCAGCTTTGGTCTC-3′, respectively. PCR products were subcloned into the pGEM-T easy vector (Promega, Madison, WI, USA). The sequences that were the target products were confirmed by the Sanger method. Plasmids containing the cDNA fragment for *HTR1A, HTR1B, HTR2A, HTR2C, HTR3A*, and *HTR4* were amplified by PCR with an M13 primer pair. The amplicons containing the T7 and SP6 promoter sites were purified using a PCR purification kit (Qiagen, Valencia, CA, USA). The digoxigenin (DIG)-labelled sense and antisense RNA probes were prepared by in vitro transcription using a DIG RNA labelling kit (Roche, NJ).

### Sectioning and in situ hybridisation

The frozen brain blocks were cut into 18 µm-thick sections using a cryostat (Leica CM3050S or Leica CM1850, Leica Biosystems, Nußloch, Germany). Serial coronal sections were prepared from level A 14.4 to A 5.6 of the Kuenzel and Masson’s atlas^32^. In situ hybridisation was performed as described previously with some modifications^60,61^. Briefly, brain sections were re-fixed in 4% PFA-PBS and pre-treated and hybridised with DIG-labelled riboprobes at 70 °C. After stringent washes, hybridised probes were detected immunohistochemically with alkaline phosphatase-conjugated anti-DIG antibody (1:1,000; Roche, NJ). To visualise the signals, a chromogenic reaction with a nitro blue tetrazolium/5-bromo-4-chloro-3-indolyl phosphate was performed at room temperature for the following duration: *HTR1A*, 15.5–38 h; *HTR1B*, 14.5–17 h; *HTR2A*, 15.5–39 h; *HTR2C*, 15.5–39 h; *HTR3A*, 13–37.5 h; and *HTR4*, 15.5–37.5 h. In every experiment, sense probes were used as negative controls.

### Imaging and data processing

Digital photographs of all brain sections on each slide glass were obtained semi-automatically with NanoZoomer 2.0HT or NanoZoomer XR systems (Hamamatsu Photonics, Shizuoka, Japan). The microscopic fields of interest were cropped using NDP.view2 software (ver. 2.7.25, https://www.hamamatsu.com/, Hamamatsu Photonics, Shizuoka, Japan). The entire cropped images were converted to 8-bit, and the brightness and contrast of the entire cropped images were adjusted using ImageJ (https://imagej.nih.gov/ij/).

## Supplementary Information

Supplementary Information.

## Data Availability

The datasets generated and/or analysed during the current study are available from the corresponding author upon reasonable request.
