# Supplementary material for: The dorsal arcopallium of chicks displays the expression of orthologs of mammalian fear related serotonin receptor subfamily genes
Source: Sci Rep. 2020 Dec 3;10:21183. doi: 10.1038/s41598-020-78247-9 (PMC7712838; doi:10.1038/s41598-020-78247-9)
Supplement: Supplementary file 1 — Supplementary Information. [file 41598_2020_78247_MOESM1_ESM.pdf]

Supplementary information to

The dorsal arcopallium of chicks displays the expression of orthologs of mammalian fear related serotonin receptor subfamily genes

Authors:

Toshiyuki Fujita[1], Naoya Aoki[1], Chihiro Mori[1], Eiko Fujita[1], Toshiya Matsushima[2], Koichi J. Homma[1], and Shinji Yamaguchi[1]*

Affiliations:

[1] Faculty of Pharmaceutical Sciences, Department of Life and Health Sciences, Teikyo University, 2-11-1 Kaga, Itabashi-ku, Tokyo, 173-8605, Japan

[2] Department of Biology, Faculty of Science, Hokkaido University, Hokkaido 060-0810, Japan

*Corresponding author:

Shinji Yamaguchi, Ph.D.

E-mail: shinji-y@pharm.teikyo-u.ac.jp

Tel: +81-3-3964-8136

Fax: +81-3-3964-8415

List of supplementary material:

Supplementary Figure Legends

Supplementary Figure 1

Supplementary Figure 2

Supplementary Figure 3

Supplementary Figure 4

Supplementary Table

Supplementary Figure 1. *In situ* hybridisation of *5-HTR1A* in the P1 chick brains
DIG-labelled RNA antisense (a-f) and sense (a'-f') *5-HTR1A* probe was used for *in situ* hybridisation in P1 chick brain coronal sections. For *5-HTR1A*, sections of five chicks were analysed and representative images of three chick brain sections are shown. (a''-f'') Diagrams of coronal sections are shown on the rightmost panels. The levels of the sections (A12.8 to A5.8) are in accordance with the chick atlas by Kuenzel and Masson [32]. A, arcopallium; Aph, area parahippocampalis; H, hyperpallium; Hp, hippocampus; LSt, lateral striatum; M, mesopallium; N, nidopallium. Scale bar = 2.5 mm.

Supplementary Figure 2. *In situ* hybridisation of *5-HTR2A* in the P1 chick brains
DIG-labelled RNA antisense (a-f) and sense (a'-f') *5-HTR2A* probe was used for *in situ* hybridisation in P1 chick brain coronal sections. For *5-HTR2A*, sections of five chicks were analysed and representative images of three chick brain sections are shown. (a''-f'') Diagrams of coronal sections are shown on the rightmost panels. The levels of the sections (A13.6 to A5.8) are in accordance with the chick atlas by Kuenzel and Masson [32]. A, arcopallium; Aph, area parahippocampalis; H, hyperpallium; Hp, hippocampus; LSt, lateral striatum; M, mesopallium; N, nidopallium. Scale bar = 2.5 mm.

Supplementary Figure 3. *In situ* hybridisation of *5-HTR3A* in the P1 chick brains
DIG-labelled RNA antisense (a-f) and sense (a'-f') *5-HTR3A* probe was used for *in situ* hybridisation in P1 chick brain coronal sections. For *5-HTR3A*, sections of eight chicks were analysed and representative images of three chick brain sections are shown. (a''-f'') Diagrams of coronal sections are shown on the rightmost panels. The levels of the sections (A13.0 to A5.8) are in accordance with the chick atlas by Kuenzel and Masson [32]. A, arcopallium; Aph, area parahippocampalis; Cdl, area corticoidea dorsolateralis; H, hyperpallium; Hp, hippocampus; LSt, lateral striatum; M, mesopallium; N, nidopallium. Scale bar = 2.5 mm.

Supplementary Figure 4. *In situ* hybridisation of *5-HTR4* in the P1 chick brains
DIG-labelled RNA antisense (a-f) and sense (a'-f') *5-HTR4* probe was used for *in situ* hybridisation in P1 chick brain coronal sections. For *5-HTR4*, sections of six chicks were analysed and representative images of four chick brain sections are shown. (a''-f'') Diagrams of coronal sections are shown on the rightmost panels. The levels of the sections (A13.6 to A5.6) are in accordance with the chick atlas by Kuenzel and Masson [32]. A, arcopallium; Aph, area parahippocampalis; H, hyperpallium; Hp, hippocampus; LSt, lateral striatum; M, mesopallium; N, nidopallium. Scale bar = 2.5 mm.

Supplemental Figure 1

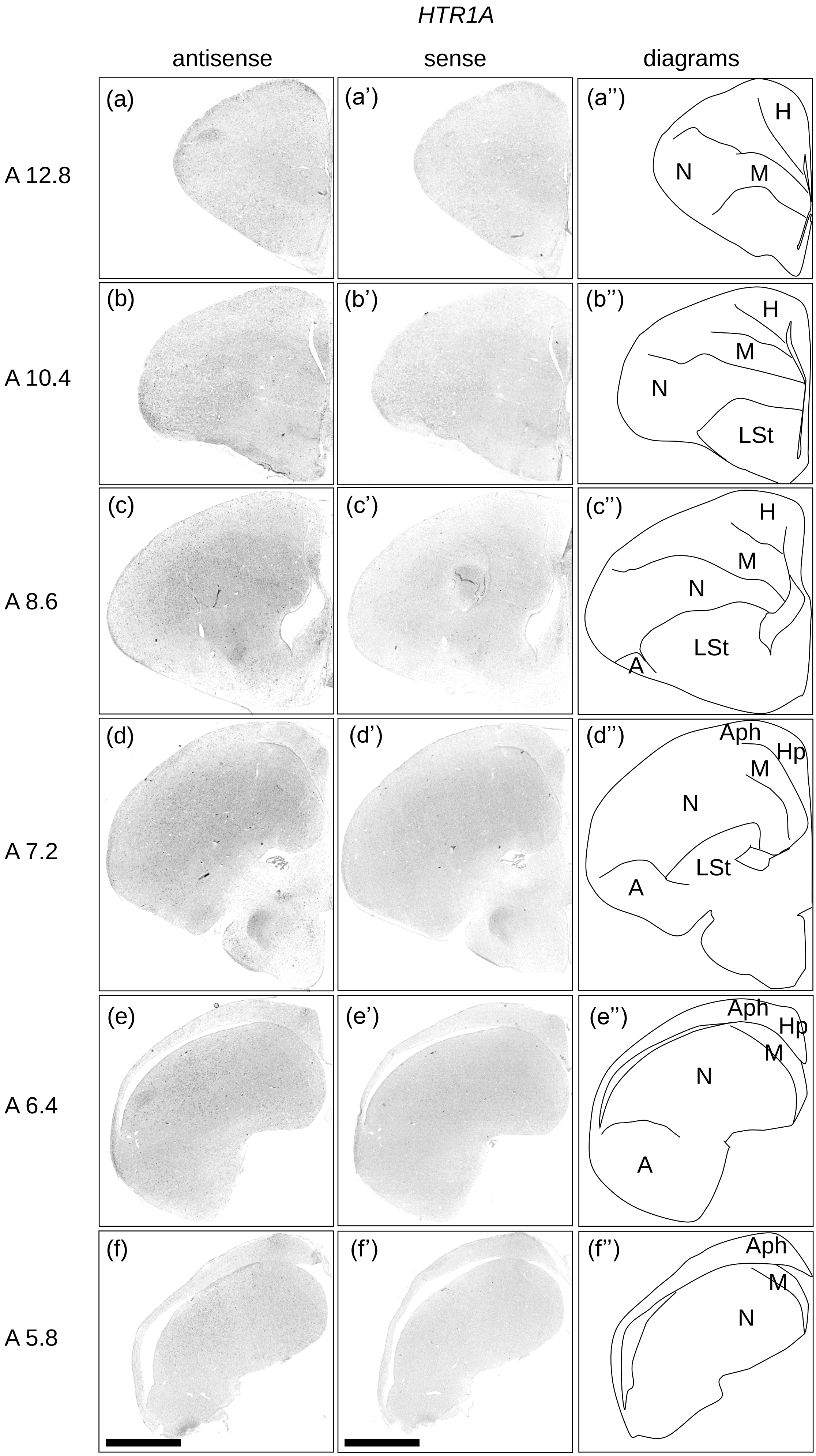

Supplemental Figure 2

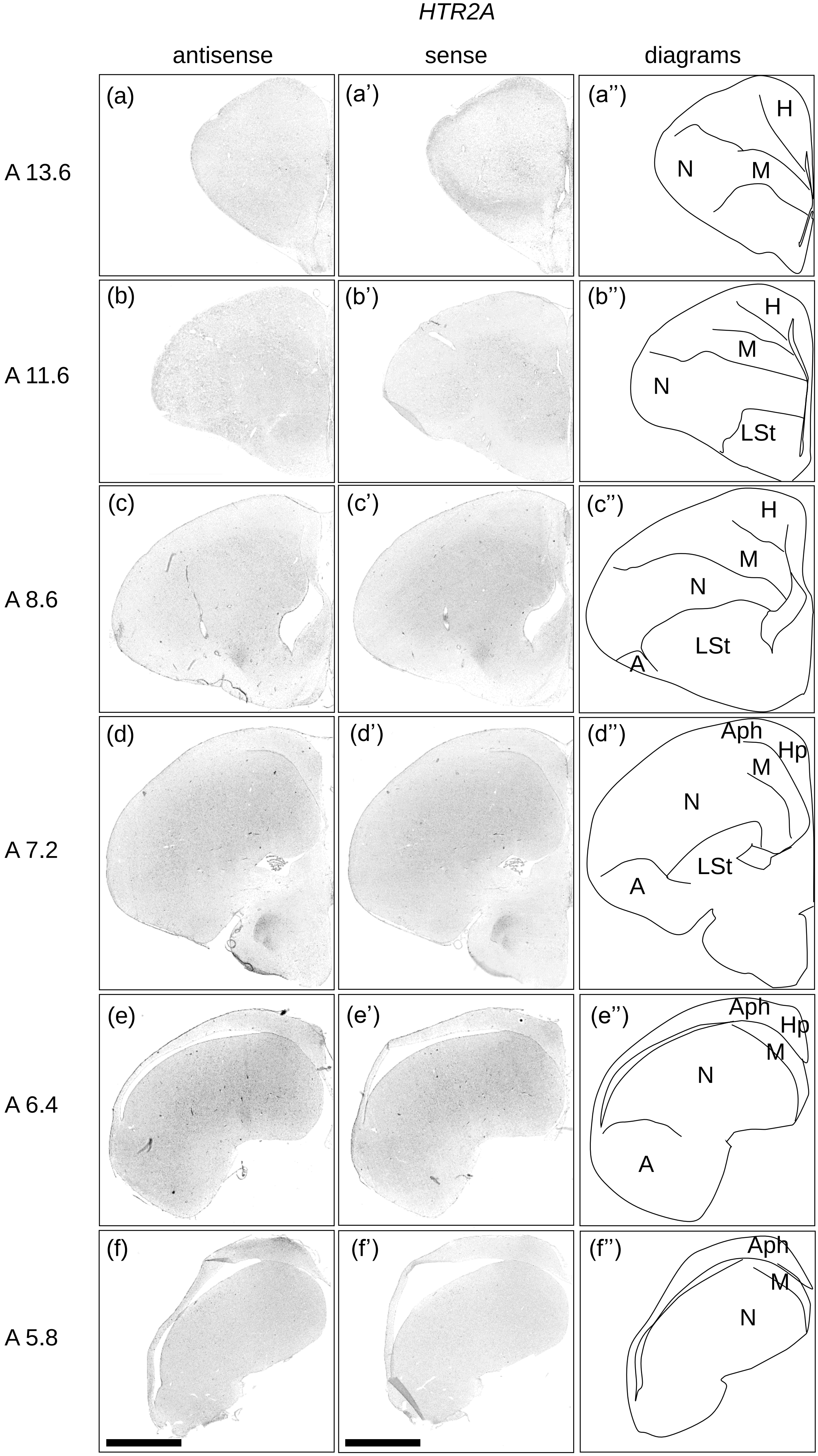

Supplemental Figure 3

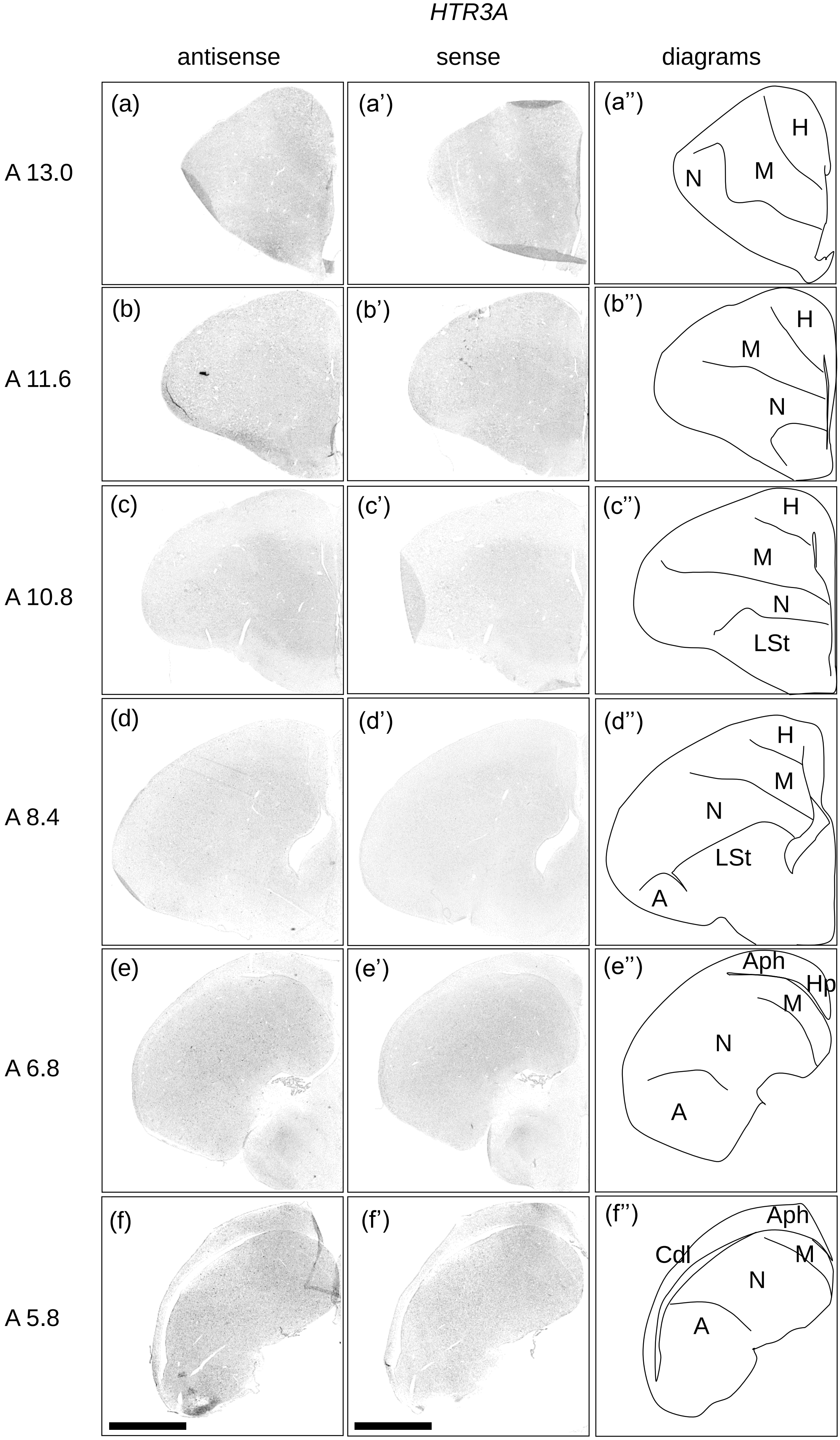

Supplemental Figure 4

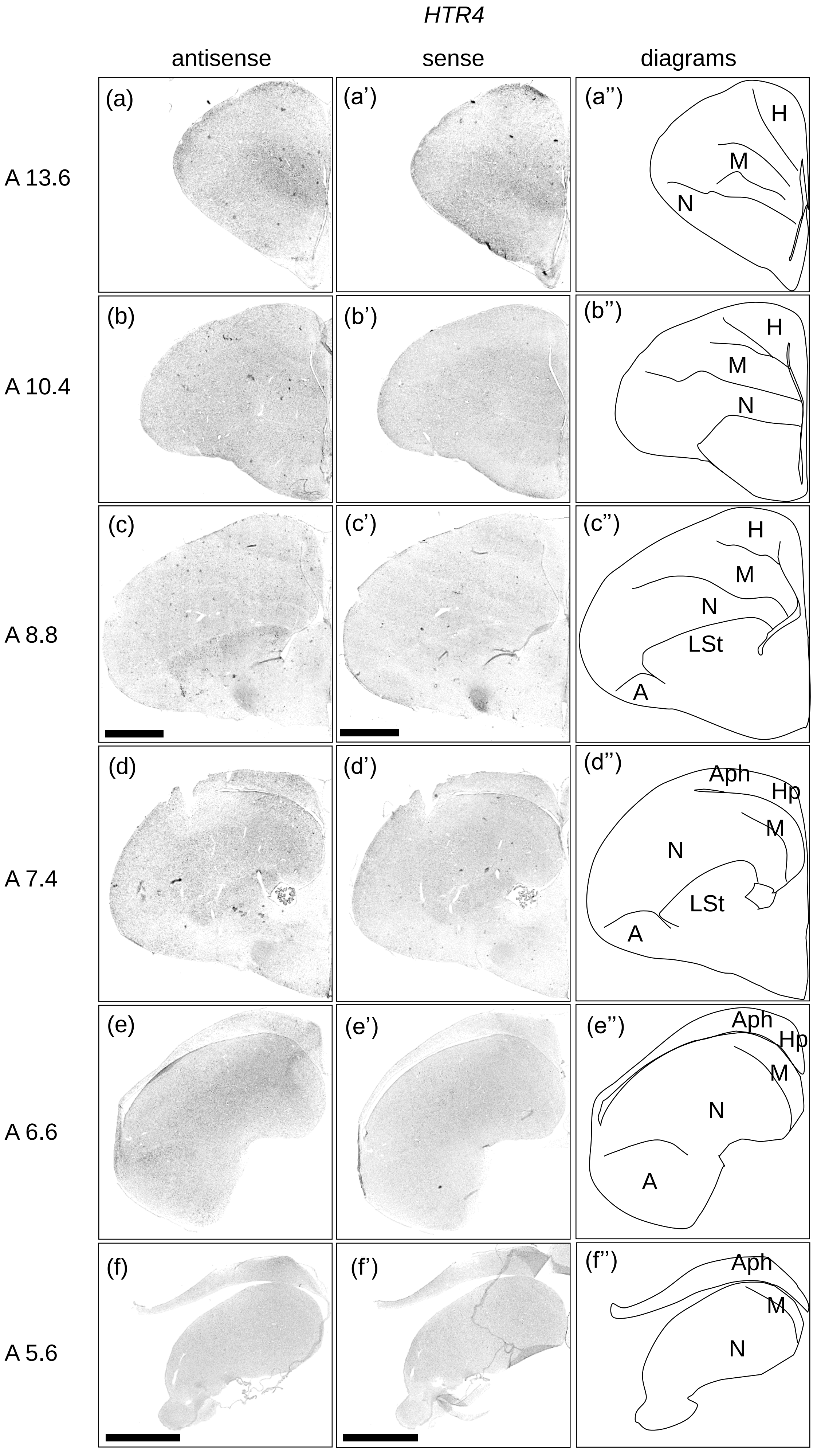

Supplemental Table 1. Subject summary used in this study

	chick #s										
	#1	#2	#3	#4	#5	#6	#7	#8	#9	#10	#11
probes											
5-HTR1A	○	○	○	○	○	-	-	-	-	-	-
5-HTR1B	○	○	○	○	○	○	○	○	-	-	-
5-HTR2A	○	○	○	○	○	-	-	-	-	-	-
5-HTR2C	○	○	○	○	○	-	-	○	○	-	-
5-HTR3A	-	○	○	○	○	-	○	-	○	○	○
5-HTR4	-	○	○	○	○	-	○	-	○	-	-
